# Brain-Tropic Metastasis: Mechanistic Checkpoints Governing Cerebral Colonization

**DOI:** 10.1159/ocl/aebag007

**Published:** 2026-08-26

**Authors:** Henry Freeman, Clayton Rawson, Denise Baloi, Michael Karsy, Mehrdad Pahlevani

**Affiliations:** a Michigan State University, East Lansing, MI, USA; b Noorda College of Osteopathic Medicine, Provo, UT, USA; c College of Human Medicine, Michigan State University, Grand Rapids, MI, USA; d Department of Neurosurgery, University of Michigan Medicine, Ann Arbor, MI, USA; e Department of Neurosurgery, Jamaica Hospital Medical Center, New York, NY, USA

**Keywords:** Blood-tumor barrier, Brain metastasis, Endothelial adhesion, Extracellular vesicle, Organotropism, Microglia

## Abstract

**Background:**

Brain metastasis is a major contributor to morbidity in advanced cancer, with substantial variability in incidence and biological behavior across primary tumor types and molecular subtypes. Increasing evidence suggests that cerebral organotropism is driven by coordinated interactions between tumor-intrinsic programs and the specialized brain microenvironment. This review aimed to synthesize contemporary mechanistic evidence underlying brain-tropic metastasis and identify biologically actionable bottlenecks involved in metastatic progression within the central nervous system.

**Summary:**

We conducted a mechanism-focused narrative review of recent neuro-oncology literature examining the biological processes that facilitate brain-specific metastatic dissemination. Four major mechanistic domains were evaluated: (1) extracellular vesicle and exosome-mediated conditioning of the brain vascular-immune niche; (2) cerebral microvascular arrest and endothelial adhesion under shear stress facilitating tumor transmigration; (3) brain-tumor barrier remodeling and endothelial-state heterogeneity influencing permeability and transport; and (4) perivascular metastatic progression shaped by astrocytic and microglial/myeloid microenvironments. The strongest mechanistic support for cerebral organotropism was identified in breast cancer, oncogene-driven non-small cell lung cancer, and melanoma. In contrast, the biological pathways underlying brain metastasis in renal cell carcinoma and colorectal cancer remain comparatively less defined.

**Key Messages:**

Cerebral organotropism appears to emerge through iterative tumor subclone evolution coupled with dynamic host microenvironmental reprogramming. Coordinated vascular, immune, and glial interactions represent critical mechanistic checkpoints governing metastatic seeding and survival in the brain. Future advances in prediction and prevention will require subtype-resolved and spatially informed human validation studies, alongside mechanistically grounded therapeutic strategies targeting vascular docking, barrier remodeling, and glial-myeloid support pathways.

## Introduction

Brain metastases (BrMs) are common complications of late-stage systemic cancer with increasing incidence given advances in local therapies and systemic agents, but with significant neurologic morbidity and mortality [1]. In a population-based analysis of >1.3 million newly diagnosed nonhematologic malignancies (2010–2013), 2.0% of all patients and 12.1% of those presenting with distant metastatic disease at diagnosis had BrMs at presentation, with marked variation by primary and histology [2]. BrMs are unevenly distributed across cancer and molecular subtypes, consistent with the known cerebral organotropism of specific tumors. The most common cerebral BrMs include non-small cell lung cancer ([NSCLC] 40–50%), breast (15–25%), melanoma (9–10%), followed by colorectal cancer and renal cell carcinomas [3]. Overall, BrMs are estimated to arise in up to approximately 30% of adults with cancer, underscoring their clinical burden [4]. Despite a high incidence, prostate cancer, hematological, pancreatic, and hepatic cancers show limited cerebral organotropism.

Considering the work of previous researchers [1, 5], we synthesized mechanistic studies from 2015 to 2025, organized evidence for brain-directed metastatic organotropism, and identified actionable checkpoints for prediction and intervention. We organized supportive evidence for a brain-specific stepwise progression framework, namely including extracellular vesicle (EV)-mediated stromal priming, cerebral microvascular arrest, and stable adhesion of tumor cells under shear, brain-tumor barrier (BTB) modulation, and perivascular niche adaptation via vessel co-option and glial/myeloid support (Fig. 1). At each pathological rate-limiting step or chokepoint, we have identified features for disease prognostication and therapeutic targets. We searched PubMed/MEDLINE and Scopus for English-language articles published between January 2015 and December 2025, using combinations of Medical Subject Headings (MeSH) terms and free-text keywords related to brain metastasis and organotropism, EVs and exosomes, cerebral endothelial adhesion, blood-brain barrier (BBB) and blood-tumor barrier (BTB) biology, vascular co-option, and central nervous system (CNS) stromal and immune interactions (for example, “brain metastasis” AND [“extracellular vesicles” OR “exosomes” OR “organotropism” OR “blood-tumor barrier” OR “vascular co-option” OR “astrocyte” OR “microglia”]). Reference lists of key reviews and high-impact primary studies were hand-searched to capture mechanistic work not retrieved by keyword searching, and foundational studies published before 2015 were included where necessary for conceptual context. Because this is a mechanism-focused narrative review rather than a systematic review, studies were selected purposively to prioritize causal and bottleneck-resolving evidence for each checkpoint; accordingly, we did not apply formal PRISMA screening, tabulate screened-versus-included study counts, or perform quantitative synthesis.

**Fig. 1.**
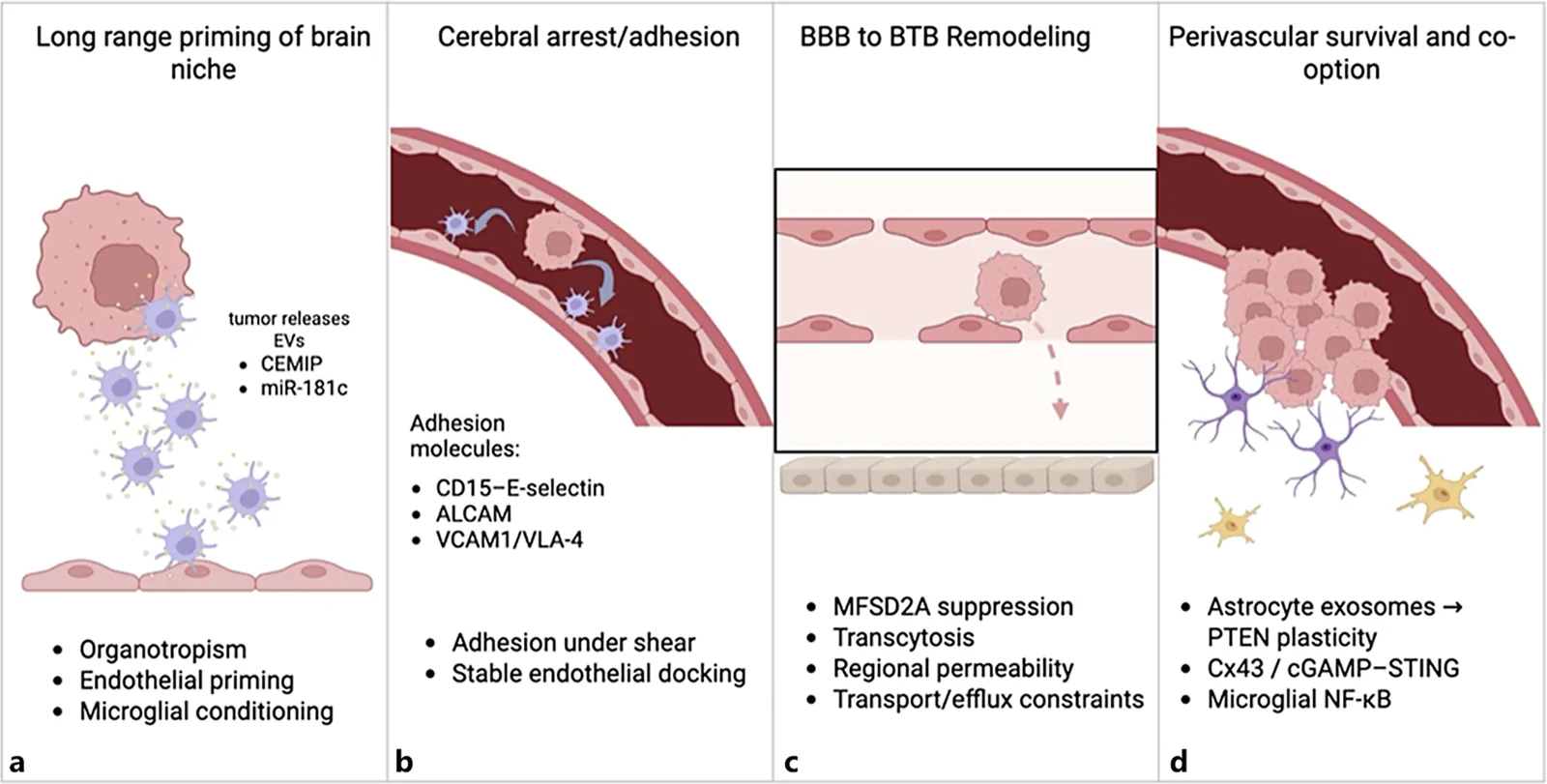
Brain-specific metastatic bottlenecks governing successful brain colonization. Brain metastasis (BrM) is conceptualized as a multistep, probabilistic process constrained by a small set of brain-specific, rate-limiting checkpoints **a** Primary tumors can prime the brain vascular-immune niche at long range by releasing extracellular vesicles (EVs) containing brain-tropic cargo (e.g., CEMIP, miR-181c), thereby activating endothelia and conditioning microglia prior to tumor cell arrival. **b** Circulating tumor cells must undergo stable arrest and adhesion within the cerebral microvasculature under physiological shear stress, mediated by specific adhesion programs (e.g., CD15s-E-selectin, ALCAM, VCAM1/VLA-4) **c** Tumor entry occurs across a remodeled blood-tumor barrier (BTB) that is spatially heterogeneous and retains active transport and efflux constraints, which are influenced by endothelial state changes, such as suppression of MFSD2A and altered transcytosis. **d** Following entry, durable metastatic outgrowth depends on perivascular survival strategies and co-option of CNS-specific support circuits, including vascular co-option, astrocyte-mediated signaling (e.g., exosome-driven PTEN plasticity, Cx43/cGAMP-STING communication), and microglial state programs (e.g., NF-κB activation). Together, these bottlenecks explain why only select tumor lineages and subclones repeatedly succeed in establishing clinically relevant BrM. ALCAM, activated leukocyte cell adhesion molecule; BBB, blood-brain barrier; CD15 s, sialyl Lewis X; cGAMP, cyclic GMP-AMP; CEMIP, cell migration-inducing and hyaluronan-binding protein; CNS, central nervous system; Cx43, connexin 43; MFSD2A, major facilitator superfamily domain containing 2A; miR, microRNA; NF-κB, nuclear factor kappa B; PTEN, phosphatase and tensin homolog; STING, stimulator of interferon genes; VCAM1, vascular cell adhesion molecule 1; VLA-4, very late antigen-4 (integrin α4β1).

## Mechanistic Synthesis: Brain-Specific Checkpoints

### EV-Mediated Long-Range Priming of a Brain-Permissive Vascular-Immune Niche

Tumor-derived EVs increase the probability of brain colonization by preconditioning components of the neurovascular unit and innate immune compartments, biasing recipient cells toward inflammatory and remodeling states that facilitate vascular capture, barrier negotiation, and perivascular survival [1, 6] (Fig. 1a). EVs are a heterogeneous population of lipid bilayer-enclosed particles; the exosome subset (endosomally derived, approximately 30–150 nm in diameter) can carry protein, mRNA, and microRNA (miRNA) cargo capable of modulating both nearby and distant microenvironments. Organotropism studies demonstrate nonrandom EV uptake, with brain-tropic exosomes preferentially localizing to cerebral endothelium and exhibiting distinct integrin profiles linked to brain-directed targeting [7].

Cargo-driven effects provide a mechanistic link between EVs and distal intracranial microenvironment modulation that is critical to tumor seeding. EV-delivered cell migration-inducing and hyaluronan-binding protein (CEMIP) promotes early brain colonization and induces endothelial and microglial remodeling [8], while EV-associated miR-181c disrupts endothelial tight junctions through cytoskeletal reprogramming, increasing BBB permeability and metastatic incidence [9]. Together, these data support a diverse model in which EVs contribute to brain metastasis through endothelial uptake and cargo-mediated reprogramming of vascular and immune niches.

### Cerebral Microvascular Arrest and Adhesion: The Brain Entry Checkpoint

Stable microvascular arrest and adhesion under shear represent early, rate-limiting steps in brain metastasis [1, 10] (Fig. 1b). This process is not purely mechanical; adhesion initiates reciprocal signaling that facilitates transmigration, reflecting molecular matching between tumor adhesion programs and cerebral endothelium. Tumor cell transmigration functions through regulated molecular mechanisms resulting in alteration of cell integrin, and cytoskeletal structure as well as direct contact and cytokine-mediated alteration of endothelial tight junctions.

In NSCLC, tumor necrosis factor α (TNF-α)-induced endothelial CD62E enhances tumor adhesion under flow, with CD15(s)-CD62E interactions mediating docking [11, 12]. Also in NSCLC, activated leukocyte cell adhesion molecule promotes endothelial adhesion and brain metastasis formation [13]. In vitro models of breast cancer adhesion to endothelium under flow conditions, selective clones with increased potential for transmigration capacity and specific upregulated adhesion molecules (i.e., MUC1, VCAM1, and VLA-4) could be identified [14]. Collectively, these findings indicate that cerebral docking is both selectable and lineage-dependent, arguing against a single universal pathway and supporting tumor-specific targeting strategies.

### Brain-Tumor Barrier Remodeling and Heterogeneous Transport Constraints

While an intact BBB aims to prevent metastatic deposits, a spectrum of remodeling of the BTB enables metastatic colonization [5, 15–17] (Fig. 1c). Permeability is influenced distinctly by tumor types and subtypes, reflecting neurovascular unit remodeling and endothelial-state changes. Experimental and imaging studies demonstrate that some metastases remain minimally permeable despite established disease [18, 19]. One mechanism of endothelial reprogramming includes suppression of Major Facilitator Superfamily Domain-containing protein 2A (MFSD2A) which alters transcytosis and barrier properties [18–20]. Importantly, increased permeability does not ensure drug delivery, as transporter-mediated efflux and preserved BBB regions limit intratumoral exposure [21]. Clinical data support biochemical heterogeneity at the BTB, and vascular co-option can preserve BBB-like features at tumor margins, reinforcing spatially patterned transport constraints [22, 23]. Thus, BTB remodeling functions as both an entry and persistence bottleneck that shapes metastatic fitness and therapeutic response.

### Perivascular Survival and CNS Niche Adaptation: Vascular Co-Option, Astrocyte Circuitry, and Microglial State

Following intracerebral entry, most disseminated cells fail to survive, and durable outgrowth depends on adaptation to a perivascular niche dominated by astrocytes and microglia [1] (Fig. 1d). Vascular co-option enables tumor cells to grow along existing microvessels through integrin-dependent interactions and cytoskeletal programs, supporting early expansion. A foundational link between perivascular survival and vascular co-option is provided by L1 cell adhesion molecule (L1CAM): metastatic cells deploy L1CAM to spread along and grow on the abluminal surface of pre-existing brain capillaries. Reactive brain stroma generates plasmin, which cleaves membrane-bound astrocytic FasL into a paracrine death signal and inactivates L1CAM; brain-metastatic breast and lung cancer cells counter this defense by expressing anti-plasminogen-activator serpins (e.g., neuroserpin, serpin B2), providing a unifying mechanism for cancer cell survival and vascular co-option in the brain [24]. Astrocytes provide critical survival signals through multiple mechanisms. Astrocyte-derived exosomal miR-19a suppresses expression of the tumor suppressor phosphatase and tensin homolog in metastatic tumor cells, a microenvironmentally induced and reversible loss that increases secretion of the chemokine CCL2, recruiting IBA1+ myeloid cells that reciprocally promote tumor cell proliferation and reduce apoptosis to prime brain metastatic outgrowth [25]. Tumor-cell protocadherin 7 (PCDH7) further promotes assembly of carcinoma-astrocyte gap junctions by recruiting connexin 43 (Cx43) [26]. Connexin 43 gap junctions can mediate cGAMP transfer from tumor cells to normal cells, activating Stimulator of Interferon Genes (STING) signaling and enhances inflammatory signaling (e.g., TNF, interferon α) that promote tumor growth [26]. One marker of reactive astrocytes, STAT3, can contribute to an immunomodulatory niche required for progression [27]. Microglial state programs also shape metastatic fitness. NF-κB-dependent microglial activation promotes tumor growth and modulates immunotherapy response, highlighting the importance of CNS-resident immune circuits in post-entry selection [28]. Together, these findings support a model in which successful colonization is determined largely after entry through niche co-option and microenvironmental reprogramming (Table 1).

**Table 1.** Mechanistic modules underlying brain-tropic metastatic propensity: evidence strength across tumor types (2015–2025)

Mechanistic checkpoint/module	Representative micro-molecular determinants (examples)	Evidence strength			
		breast (TNBC/HER2+)	NSCLC (EGFR-mut/ALK+)	melanoma	RCC/CRC (comparators)
EV-mediated brain priming (pre-metastatic niche)	Exosomal integrins (organotropism); CEMIP; miR-181c	Strong	Strong	Moderate	Negative-space comparators. Both disseminate systemically yet seed the brain inefficiently, and no brain-specific program is established at any checkpoint (limited, indirect, or correlative evidence only). Their low CNS tropism most plausibly reflects failure at cerebral docking and/or post-entry niche adaptation rather than at dissemination, helping isolate genuinely brain-specific requirements from generic metastatic competence
Cerebral microvascular arrest and adhesion	CD15s-E-selectin; ALCAM (CD166); MUC1/VCAM1/VLA-4	Moderate	Strong	Emerging	
BBB-to-BTB remodeling and transport constraints	BTB heterogeneity (permeability/efflux); endothelial MFSD2A/Mfsd2a suppression	Strong	Strong	Moderate	
Perivascular survival and vascular co-option	Integrins; L1CAM; vessel co-option programs	Moderate	Moderate	Moderate	
Astrocyte support circuits	Astrocyte exosomal miR-19a → PTEN plasticity/CCL2; PCDH7/Cx43 gap junction (cGAMP-STING); STAT3+ reactive astrocytes	Strong	Strong	Moderate	
Microglial and myeloid state programs	Microglial RelA/NF-κB; myeloid recruitment/education (e.g., CCL2-linked)	Moderate	Moderate	Strong	

Evidence grading: Strong indicates multiple independent studies with causal/functional evidence in brain-metastasis-relevant models and/or human corroboration. Moderate indicates at least one causal/functional study with supportive corroboration. Emerging indicates limited, indirect, or primarily correlative evidence in brain-metastasis contexts [4, 5, 7–14, 20, 23, 25–28]. RCC and CRC are presented as negative-space comparators rather than graded per checkpoint: they metastasize widely but reach the brain infrequently, so the absence of an established brain-specific mechanism at each checkpoint is itself informative and motivates the lineage-specific predictions developed in the text [1, 4, 10].

EV, extracellular vesicle; BBB, blood-brain barrier; BTB, blood-tumor barrier; NSCLC, non-small cell lung cancer; RCC, renal cell carcinoma; CRC, colorectal cancer.

### Why Do Some Cancers, and Some Subtypes, Repeatedly Succeed in the Brain?

Brain-metastatic propensity can be understood as repeated success across a small set of CNS-specific checkpoints. High-liability cancers, particularly breast cancer, oncogene-driven NSCLC, and melanoma, consistently generate subclones capable of vascular capture, barrier negotiation, and niche adaptation, whereas renal cell carcinoma (RCC) and colorectal cancer (CRC) – which disseminate systemically yet seed the brain far less efficiently – appear to falter at one or more of these checkpoints rather than engaging the full program [1, 4, 10]. Used deliberately as “negative-space” comparators, these lower-liability lineages help distinguish genuinely brain-specific requirements from generic metastatic competence: their limited parenchymal tropism despite high extracranial metastatic burden points to failure at cerebral docking and/or post-entry niche adaptation rather than an inability to disseminate. This supports a checkpoint-based model in which subtype enrichment reflects differences in the frequency of checkpoint competence rather than a single “brain-tropic switch.” High-liability tumors exhibit coordinated programs spanning EV signaling, endothelial interaction, and glial engagement, while lower-liability cancers fail at dominant steps such as docking or persistence. Defining the limiting checkpoint for each lineage is therefore critical for prediction and intervention.

### Translational Implications: How to Turn Bottlenecks into Biomarkers and Strategies

A bottleneck framework suggests a pragmatic translational goal: identify measurable features that indicate which checkpoint a patient’s tumor is most likely to overcome and then target the most actionable checkpoint for prevention or treatment. EV cargoes are among the most tractable biomarker classes because they can, in principle, be sampled longitudinally. Studies identifying brain-priming cargoes (e.g., CEMIP; miR-181c) support that circulating EV profiles might help flag tumors capable of vascular-immune priming or BBB destabilization [8, 9]. The key next step is not simply detecting these signals but validating whether they add predictive value beyond clinical subtype and extracranial disease burden.

Adhesion and endothelial-interaction pathways are attractive because they sit upstream of established lesions. The CD15-E-selectin axis and activated leukocyte cell adhesion molecule illustrate that brain-endothelium docking can be mechanistically targetable in models [11, 13]. The translational challenge is specificity: many adhesion programs are shared with normal leukocyte trafficking, so the field needs strategies that reduce cerebral docking of tumor cells without broadly impairing vascular or immune homeostasis. BTB heterogeneity argues against assuming uniform intracranial drug access and supports the integration of barrier biology into trial design and response interpretation [5]. Mechanisms such as endothelial-state shifts (including Mfsd2a suppression) suggest that barrier normalization or controlled modulation might eventually become a rational adjunct, though this remains speculative and will require careful safety constraints given the BBBs essential physiologic role [5, 20].

Astrocyte and microglial circuits are compelling because they represent vulnerabilities that may be shared across multiple primaries once brain colonization begins. Melanoma models provide particularly direct evidence that microglial RelA (NF-κB p65) signaling can shape intracranial metastatic fitness and immunotherapy responsiveness, motivating exploration of microenvironment reprogramming as a brain-specific adjunct strategy [28].

### Limitations

This review is intentionally mechanistic and narrative rather than meta-analytic, and its conclusions should be interpreted in that context. By prioritizing causal and bottleneck-resolving studies, the synthesis necessarily introduces selection bias toward tumor types and pathways with deeper experimental investment, most notably breast cancer, NSCLC, and melanoma, while leaving comparator malignancies such as RCC and CRC comparatively underrepresented in brain-specific analyses.

A second limitation is model dependence. Much of the causal evidence discussed derives from experimental metastasis paradigms (e.g., intracardiac injection, brain-tropic derivatives, and immunodeficient hosts), which can compress the natural history of dissemination, alter immune selection pressures, and obscure distinctions between key stages such as seeding, extravasation, dormancy, and outgrowth. As a result, mechanistic inferences may not fully recapitulate the temporal and biological complexity of human disease.

Barrier biology introduces an additional interpretive constraint. Because the transition from the BBB to the BTB is spatially heterogeneous, many studies rely on simplified or indirect proxies of permeability and transport. These measures may not generalize across lesions, treatment conditions, or microregional niches within tumors, limiting the ability to draw unified conclusions about barrier function.

Finally, although clinical subtype enrichment motivates this review, subtype-resolved human validation remains uneven. Many proposed mechanisms, particularly those involving multicellular interactions at specialized interfaces such as perivascular regions, astrocyte endfeet, or myeloid niches, are not well captured by bulk tumor datasets, constraining the translational interpretation of these pathways.

### Gaps in the Field and Future Directions

Despite substantial progress, several key gaps continue to limit the ability to translate mechanistic insight into predictive frameworks. First, the timing and hierarchy of metastatic progression remain incompletely resolved. While discrete modules, such as EV priming, vascular adhesion and docking, BTB remodeling, vascular co-option, and astrocyte or microglial reinforcement, are increasingly well described, it is still unclear which steps are rate-limiting for specific tumor lineages or whether dominant decision points exist that determine brain metastatic potential [1, 10]. Addressing this will require checkpoint-resolved necessity mapping, in which perturbation studies are explicitly designed to interrogate distinct stages of the cascade rather than collapsing outcomes into a single metastatic endpoint.

Second, human validation remains limited and often subtype-agnostic. Although clinical patterns of subtype enrichment are well established, mechanistic confirmation in large, subtype-resolved human datasets is sparse, particularly for multicellular circuits such as astrocyte and microglial programs that operate at spatially restricted interfaces [26, 27]. Future work should prioritize paired, spatially resolved datasets spanning primary tumors, extracranial metastases, and BrMs to capture these interactions in context.

Third, BTB heterogeneity is widely recognized but inconsistently operationalized. Studies vary in how barrier disruption is measured, and mechanistic findings are often disconnected from spatial pharmacology. For example, suppression of endothelial regulators such as MFSD2A/Mfsd2a has been linked to increased permeability without classic tight junction loss, highlighting the need to integrate endothelial-state profiling with lesion-level drug exposure and intratumoral heterogeneity [5, 20]. A critical priority is to align molecular markers of barrier state with functional readouts of transport and therapeutic delivery.

Fourth, comparator tumor types remain underutilized as experimental controls. Malignancies such as RCC and CRC, which metastasize systemically but exhibit lower brain tropism, offer an opportunity to distinguish brain-specific mechanisms from general metastatic programs. Incorporating these comparators into matched assays of endothelial adhesion, BBB transmigration, and post-entry survival would strengthen causal inference and clarify lineage-specific vulnerabilities.

Looking forward, several strategies could accelerate translation. Prospective biomarker validation in high-risk subtypes, particularly involving EV cargo and endothelial-state signatures, should determine whether these features provide predictive value beyond established clinical factors. BBB heterogeneity should be explicitly integrated into study design through combined imaging, molecular profiling, and pharmacokinetic measurements. Finally, mechanism-matched therapeutic strategies should be tested in immunocompetent models with designs that distinguish effects on seeding, outgrowth, and treatment response. Longitudinal lineage tracing and single-cell profiling of early micrometastases will be essential to define when key processes such as vascular co-option, astrocyte coupling, and myeloid state transitions become dominant and which pathways govern dormancy escape.

Collectively, these efforts would enable the transition from a descriptive mechanistic catalogue to a predictive framework capable of identifying lineage-specific bottlenecks and informing targeted strategies to prevent or control brain metastasis.

## Conclusion

Over the past decade, BrM has shifted from being framed as a passive end-stage complication to being understood as a mechanistically tractable, brain-specific selection process. The evidence synthesized here supports a bottleneck model in which differential “brain preference” arises when a tumor lineage repeatedly generates subclones that can (1) prime the brain vascular-immune niche at distance (often via EV programs), (2) achieve stable cerebral microvascular arrest and adhesion under shear, (3) negotiate a heterogeneous BBB→BTB landscape with persistent transport constraints, and (4) secure durable perivascular survival by co-opting vascular, astrocytic, and myeloid circuits that reinforce growth and therapy resistance. This framework helps explain why breast cancer, oncogene-driven NSCLC, and melanoma account for a disproportionate share of clinical BrM, and it clarifies that successful colonization is often “won” after entry through niche co-option rather than access alone. The principal contribution of this synthesis is therefore conceptual: it reframes brain tropism as repeated success across a defined, ordered set of checkpoints and proposes lower-tropism malignancies such as RCC and CRC as negative-space comparators for isolating genuinely brain-specific requirements, yielding testable, lineage-specific predictions rather than a descriptive catalogue. Ultimately, converting mechanistic catalogs into predictive, subtype-resolved models, validated in spatially resolved human datasets and comparator primaries, will be essential to move from describing brain tropism to reliably predicting, preventing, and treating brain metastasis.

## Conflict of Interest Statement

Michael Karsy discloses royalties from Thieme Medical Publishing, consulting for Leica Microsystems, and grant funding for JNJ/Ethicon. The other authors have no conflicts of interest to declare.

## Funding Sources

This study was not supported by any sponsor or funder.

## Author Contributions

H.F.: conceptualization, methodology, validation, visualization, writing – original draft, review & editing. C.R.: validation, visualization, writing – original draft, review & editing. D.B.: writing – original draft. M.K.: conceptualization, methodology, project administration, resources, supervision, validation, visualization, writing – review & editing. M.P.: conceptualization, methodology, project administration, supervision, validation, visualization, writing – original draft, review, & editing.
